# Critical care paramedics’ experiences of performing an emergency scalpel cricothyroidotomy: a qualitative study

**DOI:** 10.29045/14784726.2022.06.7.1.3

**Published:** 2022-06-01

**Authors:** Duncan Aldred, Mark Durham, Nora Prokop, Gary Balderston, Richard Crabb, Paul Crouch, Lewis Pike, John Children, Andy McBride, Adam Heywood, Julia Williams, Alan Cowley

**Affiliations:** South East Coast Ambulance Service NHS Foundation Trust; South East Coast Ambulance Service NHS Foundation Trust; South East Coast Ambulance Service NHS Foundation Trust; South East Coast Ambulance Service NHS Foundation Trust; South East Coast Ambulance Service NHS Foundation Trust; South East Coast Ambulance Service NHS Foundation Trust; South East Coast Ambulance Service NHS Foundation Trust; South East Coast Ambulance Service NHS Foundation Trust; South East Coast Ambulance Service NHS Foundation Trust; South East Coast Ambulance Service NHS Foundation Trust; South East Coast Ambulance Service NHS Foundation Trust ORCID iD: https://orcid.org/0000-0003-0796-5465; South East Coast Ambulance Service NHS Foundation Trust ORCID iD: https://orcid.org/0000-0002-3093-4395

**Keywords:** critical care, pre-hospital, specialist paramedic

## Abstract

**Introduction:**

A scalpel cricothyroidotomy or front of neck access (FONA) is a rarely performed part of airway management for when other steps have failed and the patient cannot be intubated or ventilated. Increasingly advanced and specialist paramedics are being trained to perform this procedure within the pre-hospital environment.

**Methods:**

Advanced and specialist paramedics within a UK ambulance service that had performed a FONA were invited to participate in this qualitative research. Semi-structured interviews were used to gather information on the participants’ experiences. This information underwent thematic analysis to develop codes which were then grouped into themes.

**Results:**

Seven participants were interviewed between December 2020 and January 2021. Three main themes were identified: the procedure, isolation and training. The main complications described were bleeding in excess of expectations, moving structures, surgical emphysema and a false track.

**Conclusion:**

Complications appeared common; training to perform a FONA should include complications and an approach to their management similar to other airway management procedures. Isolation was a common theme within this study, however remote support from a peer appeared beneficial.

## Introduction

Airway management is a fundamental principle of pre-hospital care and, while rare, the ‘cannot oxygenate, cannot ventilate’ situation is an immediately life-threatening emergency where all established steps have failed and the patient is not receiving any ventilation. In keeping with the structured airway management ladder as described in the Difficult Airway Society (DAS) guidelines (Frerk et al., 2015), a scalpel cricothyroidotomy or ‘front of neck access’ (FONA) should be performed.

South East Coast Ambulance Service NHS Foundation Trust (the Trust) has developed the critical care paramedic (CCP) role to provide additional advanced pre-hospital care. CCPs comprise experienced paramedics who have undertaken postgraduate study, including in-hospital and work-based placements. They have 24-hour access to advice from a remote consultant physician to support with patient treatment and management.

These CCPs are trained to perform a FONA as part of airway management. The FONA theory, training and practice is led by a consultant-level clinician. Initial training utilises sheep larynges, with continued training using commercially purchased training aids. Additionally, CCPs undertake clinical governance every 7 weeks. This allows incidents to be reviewed and debriefed both with their peers and with one of the Trust’s consultant physicians. Each governance cycle will also require CCPs to undertake a simulated FONA overseen and critiqued by their peers to ensure competence is maintained.

When a CCP performs a FONA, the incident is de-briefed with the Trust’s consultant physicians and CCP management team followed by peer-to-peer discussion within their governance team.

The Trust covers an area of 3600 square miles in which the population varies between dense urban areas and sparse rural areas. The Trust has over 4000 staff, over 110 sites (South East Coast Ambulance Service NHS Foundation Trust, 2020).

Within the Trust area, between 19 August 2017 and 31 May 2021, CCPs attended 6736 out-of-hospital cardiac arrests but were only required to perform a total of nine FONAs. Within the FONAs reviewed in this study, two involved choking, one trauma, one body habitus with previous difficult airway, two burns and a further case involved burns with other complications.

The aim of this study is to explore CCPs’ experiences of performing a FONA in the pre-hospital arena and to identify any lessons that can be learned. It is hoped that by sharing this information, practice can be improved, and clinicians will be better prepared to undertake future FONAs.

## Methods

### Recruitment and selection

The inclusion criterion was that participants had to be a CCP who had performed a FONA (from its introduction to the Trust in November 2015 to October 2020) while working as a CCP for the Trust. An email was sent to all current CCPs within the Trust inviting them to participate if they met this criterion. Those eligible and willing to be involved were then contacted to arrange an interview. One of the authors was also interviewed as a participant, but they were not involved in the thematic analysis stages to reduce the chance of bias within that stage of the study.

### Data collection

Semi-structured interviews, lasting on average 42 minutes (31, 36, 39, 41, 42, 48, 56 minutes), were used to gather data. The initial structure for the interviews was derived from the existing knowledge and experience of the authors. The content of the interviews was allowed to evolve iteratively during the course of the study. To help ensure the initial interview schedule addressed the research question, two pilot interviews were first conducted with CCPs who had not been involved in the study design up to this point, and who had just undergone a high-fidelity FONA training scenario.

Interviews were all conducted by the lead researcher (DA), a male CCP who holds a PgCert and has 12 years’ experience in pre-hospital critical care. This individual has a working relationship with the subjects, all of whom are colleagues (and peers). A single interviewer was chosen to ensure consistency of approach, and interviews were conducted and recorded via Microsoft Teams^TM^. On each occasion, only the interviewer and participant were present on the video call and in the rooms. Interviews were transcribed by the authors, and double checked for accuracy. Recordings were then deleted prior to coding, to protect anonymity. There was no requirement for any repeat interviews. Data saturation was not anticipated due to the relatively small number of participants, and it was not felt this was reached during the interview process.

### Data analysis

Braun and Clarke’s (2006, 2019) model for thematic analysis was selected for the freedom and versatility it offered, within an effective structure, to support depth of findings while retaining ease of use.

Coding was performed manually by three authors (DA, MD and NP). This was first done independently of one another, then collaboratively to optimise the dependability and consistency of coding. This approach fostered reflexivity in the thematic analysis; the coding framework evolved and became more refined as the process continued and the authors discussed different aspects. Indexing during this stage was done using Microsoft Word^TM^ and a non-proprietary add-on (Fredborg, 2020), and Microsoft Excel^TM^. The coding stage was deemed complete once the three authors agreed that the framework was as accurate and thorough as possible and encompassed each of the interviews equally well. Two other authors (AH and RC) then member-checked the framework to further ensure dependability.

After coding, themes were cultivated by successive mind-mapping until a final mind map was agreed upon which best encompassed and expressed the coding framework. This was then ‘code weaved’ into prose in the manner described by Saldana (2021). A final member-check was provided by the remaining authors when they assessed the code-weaved prose in the context of the original transcripts. The transcripts were not returned to the original participants at any point.

## Results

Of the nine CCPs contacted, seven agreed to participate, while two did not respond to the email and so were assumed to have declined. No reason was given by the two individuals. Demographically, the seven CCPs comprised two females and five males, which is in proportion to the gender split in the CCP programme. The mean average age of participants was 42 years (SD 6.7).

Three main themes emerged from the interviews: the procedure, isolation and training involved in FONA.

### Procedure

Five of the participants identified early on that their patient was likely to progress to a FONA. This recognition generally happened during the primary survey or initial laryngoscopy attempt. Although the majority of the participants report identifying early that the patient may require a FONA, the decision-making process differed between participants. Five participants used a stepwise approach as described by the DAS guidelines whereas two participants adopted a more heuristic, and less algorithmic, approach to airway management.

All the participants verbalised an airway emergency and stated they were moving on to a surgical airway to try to ensure that the whole team was aware what was happening and that everyone shared the same mental model. One of the participants reflected whether they should have explained the process to the team in further detail to ensure they were prepared and understood what a surgical airway involved. Two of the participants described working through the airway algorithm as a way of allowing them to mentally prepare for the FONA after early recognition of a FONA being a likely endpoint.

Four participants described committing to the procedure and making the initial incision as the hardest part, with the location providing a mental barrier.

*The fact that it is somebody’s throat. I think that makes a difference for me.* (Participant 3)

Following the initial incision, the procedure tended to be described as physical steps unless difficulties were encountered. All the participants used an initial horizontal incision, even when landmarks could not be palpated. Four participants reported then moving to a vertical incision, allowing them to palpate for landmarks; this could be related to pre-conceived expectations from training using a model with clear landmarks. Tactile feedback to locate landmarks or to overcome complications was used by six participants, differing from training.

*pulled the stab scalpel out my little finger went in just instinctively.* (Participant 6)

The main complication encountered was blood in excess of expectations. This often caused doubt or worry with the participants.

*you cut and all this blood starts coming out, and you’re desperately trying to make it look like this is really normal.* (Participant 1)

*There was no ‘oh look there’s the cricoid thyroid membrane’. There was fatty tissue, there was blood and there was palpation and that was it. I couldn’t use my eyes to place anything, I had to feel.* (Participant 7)

Three participants mentioned that the FONA equipment only contained one pack of swabs, which they felt was insufficient to manage the bleeding. These participants also felt that the presence of a single pack of gauze had led them to expect less bleeding than encountered. In only two cases the volume of blood was not mentioned; these both involved burns. In one case involving burns, the skin was taut and did not stretch as expected; in this case tracheal dilators were useful to overcome this complication. Three participants mentioned tissues moving or being more malleable than expected. Surgical emphysema occurred in two cases. One participant found a false track but removed the endotracheal tube and used tactile feedback from their finger to identify the correct location and placed the bougie alongside. In two cases the participant was unable to ventilate the patient.

Four participants reported experiencing the effects of the adrenalised nature of the incident once the procedure had been completed or after the resuscitation had stopped.

### Isolation

Generally, the participants expressed the feeling of pressure during the incident at some point. All the participants mentioned elements related to time criticality and the pressure to perform the FONA while being aware of timescales. Five participants described a feeling of apprehension building up to committing to a FONA, with one participant describing how declaring an airway emergency was accepting and committing to the procedure.

Most participants described the team on-scene as being helpful and supportive. Different grades of staff were allocated the role of assistant and were all described as being supportive and efficient. Despite this, there was a strong sense of isolation described by participants.

*I felt like it was me doing it, and everyone else was staring.* (Participant 1)

Five participants suggested the isolation was related to the fact they were the only clinician on-scene able to perform a FONA or that the decision making and procedure was their responsibility.

*but I still certainly felt like it was very much kind of on an island, and that decision to do it was mine and mine alone, and the consequences of it going wrong therefore, are mine and mine alone.* (Participant 1)

Of the seven participants, five used the word ‘lonely’ during the interview to describe feelings during the procedure. Within the Trust, CCPs work as single response practitioners. There were two participants who did not report feeling lonely while performing the FONA. One had repeatedly practised the procedure with a previous crew mate, who assisted the procedure. The other participant arrived at the incident where a familiar and experienced paramedic had already declared a ‘cannot intubate, cannot ventilate’ airway emergency, thus suggesting that a surgical airway would be required. Two participants described a sense of crews blindly complying; participants suggested this could be related to a situation unfamiliar to the crews.

*I could have said anything and they would have gone: ‘ok that’s fine’.* (Participant 1)

Unsuccessful FONA attempts also led to a sense of isolation. Participants mentioned how their experiences differed from what they had practised and that their training had not included the eventuality of a failed FONA.

*but we don’t expect it to fail; there isn’t a plan E. So, when you can’t secure that airway and its failing, that’s a desperate place.* (Participant 4)

Four participants found good support from contact with a CCP colleague working within the control room or talking to one of the Trust’s remote physicians.

*I was really aware of having [critical care desk operative] at the end of a phone on the CCD, and just getting advice from him … I never asked him: ‘should I do this/can I do this?’ I’d already made a decision and was going ahead.* (Participant 3)

### Training

All participants described the training provided by the service as good at a basic level.

*it’s really good in drilling the procedure. So, I was very clear on like, step one, step two, step three: cut, bougie in, tube in.* (Participant 6)

It was felt that existing training built good muscle memory through repetition and helped to simplify the decision-making process. Two participants commented on the benefit of mental rehearsal.

*the visualisation of those types of skills is really, really valuable.* (Participant 2)

Training expectations appeared to relate to the isolation experienced by participants. Although the participants recognised that their training had helped them acquire a muscle memory of the procedure, they felt underprepared to deal with complications and FONA failure.

*FONA training, we firefight it and we sort it out and we end up with a positive result. You know it always ends with us securing an airway. I don’t think we’ve ever trained for it not working at all.* (Participant 4)

*expecting it to be a straightforward procedure, so in my head I think I thought it would be quite straightforward, so I wasn’t particularly worried. There was a bit of surprise when it didn’t work, I think.* (Participant 7)

Three participants discussed how they had since altered training within their team to increase fidelity, incorporating decision making and complications. Two participants commented on the difficulties that were associated with performing a FONA in the pre-hospital setting (poor weather conditions, poor lighting, etc.). As a result, the participants reported having modified their continuing training methods to reflect the challenges of pre-hospital practice and in response to their feelings when presented with complications. Six participants stated that use of cadavers or animal anatomy may benefit training. It was felt this could provide more realism and preparation for complications that are not provided by repeating the procedure on a model.

*I was like ‘It’s got to be here somewhere, because it’s always here on that foam model’.* (Participant 5)

## Discussion

The unpredictability of pre-hospital care creates challenges that may not be encountered in other environments. Although the DAS provides a guideline for airway management (Frerk et al., 2015); as an invasive and rarely performed procedure the participants described the feeling of pressure that it created. Airway algorithms have been developed to reduce poor judgement and decision making (Yang et al., 2015); rehearsal of the DAS guideline allowed the participants to proceed to perform a FONA in a timely manner.

For the majority of participants, the events described were their first exposure to FONA on a patient; expectations would have been related to training limited to manikins and some animal models. The introduction of cadaver laboratory and tissue training was suggested by multiple participants to improve FONA training. Although tissue training has been found to be an effective training modality to reach competence (Luckey-Smith et al., 2020) and increase learner satisfaction (Kennedy et al., 2014), there is currently no high-quality evidence demonstrating a significant benefit from animal model training when compared to high fidelity simulator training (Pandian et al., 2020). The use of cadavers has been found to be superior for landmark location and tissue fidelity (Myatra et al., 2017), which would be beneficial to providers with limited surgical experience. Simulation training presents its own limits, with manikins limited to the realism and difficulties they can reproduce (Hesselfeldt et al., 2005). Benefit may be gained from using varied simulators to meet different training needs (Nargozian, 2004) and may increase the range of skills (Yang et al., 2015). Training simulators should consider the expectations of the practitioners; the use of a simple neck trainer within the CCP cohort may have led to mental challenges when presented with a patient instead of just a neck to perform the procedure. The lack of fidelity with the simulators may have also led to a lack of preparedness when it came to problem solving and complication management. Training to perform an uncomplicated FONA is not enough to prepare for real-life practice, and training should incorporate complications and adaptions to techniques like training in any airway procedure. Full-scale simulation is effective in training to manage complications (Sollid et al., 2008). The participants highlighted the importance of a shared mental model and communicating effectively with the rest of the team when performing a FONA. To improve clinical outcomes, training should also consider incorporating non-technical skills (Yang et al., 2015). The use of role play to create a crisis situation can also be useful within non-technical skill training (Myatra et al., 2017).

Alongside the prevalence of feelings of isolation, participants often described finding support and benefit from speaking to a peer or consultant after the event, even though all participants were confident in their decision making that brought them to the FONA situation. It seems reasonable to posit therefore that participants’ desire to seek out that support stemmed not from a desire to validate their decision making, but from a desire to alleviate the sense of isolation. It has been shown both in healthcare (Aira et al., 2010) and corporate settings (Rokach, 2014) that even senior staff can find it lonely in a senior position without at least peer support.

### Limitations

It is recognised that this study has some limitations. The participants were volunteers which may lead to selection bias as not all FONAs from the Trust were included. The study size is small and the single responder approach of a CCP may not translate to other areas of practice. One participant was also part of the study team; although not involved in the thematic analysis, their involvement may have biased investigating issues arising from their experience. Nevertheless, it is envisaged that there is a high degree of external validity and the themes can be transferable to other pre-hospital practitioners who have FONA within their scope of practice.

Finally, the transcripts and results were not returned to the original participants, meaning that it is not possible to verify that any comments were not misinterpreted.

## Conclusion

This qualitative analysis of seven semi-structured interviews gives an insight into the experiences and feelings of CCPs in performing a FONA.

The three main themes that emerged interlink and identify areas of practice that may be improved by changes to training. Complications encountered were blood, moving tissues, a false track and failure to ventilate the patient. The use of tactile feedback appeared common to attempt to manage complications. A sense of isolation appeared common even when colleagues on-scene were described as supportive. The participants appeared to benefit from remote contact with a peer as a source of support.

From this study, changes to training to incorporate the challenges of performing a FONA on a patient, including complications and failure to ventilate, may help to prepare CCPs and reduce feelings of isolation on-scene.

## Author contributions

All authors met the journal definition for authorship. Specifically, DA led the authorship team and established the framework for the interviews and thematic analysis. All authors except JW and AC helped to transcribe the interviews. DA, MD and NP performed the coding thematic analysis. GB, MD, LP, NP and DA led the manuscript write-up, with all authors reviewing and editing. JW and AC acted as advisors for the project and were responsible for final draft revisions and formatting. AC acts as the guarantor for this article.

## Conflict of interest

JW is on the *BPJ* editorial board.

## Ethics

The study obtained HRA approval (IRAS ID: 291721) and received internal research and development approval. A full Data Protection Impact Analysis was performed and approved by the Trust’s information governance department. This study meets the requirements of the Consolidated Criteria for Reporting Qualitative Research (COREQ) checklist.

## Funding

None.
